# Prospective Evaluation of Single Nucleotide Variants by Two Different Technologies in Paraffin Samples of Advanced Non-Small Cell Lung Cancer Patients

**DOI:** 10.3390/diagnostics10110902

**Published:** 2020-11-03

**Authors:** Elba Marin, Roxana Reyes, Ainara Arcocha, Nuria Viñolas, Laura Mezquita, Elena Gonzalvo, Karmele Saez de Gordoa, Pedro Jares, Noemi Reguart, Cristina Teixido

**Affiliations:** 1 Division of Medical Oncology, Hospital Clínic, 08036 Barcelona, Spain; elmarin@clinic.cat (E.M.); rmreyes@clinic.cat (R.R.); aarcocha@clinic.cat (A.A.); nvinolas@clinic.cat (N.V.); lmezquita@clinic.cat (L.M.); nreguart@clinic.cat (N.R.); 2 Translational Genomics and Targeted Therapeutics in Solid Tumors, Institut d’Investigacions Biomèdiques August Pi I Sunyer, 08036 Barcelona, Spain; 3 Unitat funcional de Tumors Toràcics, Hospital Clínic, 08036 Barcelona, Spain; pjares@clinic.cat; 4 Division of Pathology, Hospital Clínic, 08036 Barcelona, Spain; gonzalvo@clinic.cat (E.G.); saezdegord@clinic.cat (K.S.d.G.); 5 Molecular Biology Core Facility, Hospital Clínic, 08036 Barcelona, Spain

**Keywords:** advanced non-small cell lung cancer, molecular diagnostics, mutations, next-generation sequencing, nCounter

## Abstract

Targeted therapies are a new paradigm in lung cancer management. Next-generation sequencing (NGS) techniques have allowed for simultaneous testing of several genes in a rapid and efficient manner; however, there are other molecular diagnostic tools such as the nCounter^®^ Vantage 3D single nucleotide variants (SNVs) solid tumour panel which also offer important benefits regarding sample input and time-to-response, making them very attractive for daily clinical use. This study aimed to test the performance of the Vantage panel in the routine workup of advanced non-squamous non-small cell lung cancer (NSCLC) patients and to validate and compare its outputs with the Oncomine Solid Tumor (OST) panel DNA kit, the standard technique in our institution. Two parallel multiplexed approaches were performed based on DNA NGS and direct digital detection of DNA with nCounter^®^ technology to evaluate SNVs. A total of 42 advanced non-squamous NSCLC patients were prospectively included in the study. Overall, 95% of samples were successfully characterized by both technologies. The Vantage panel accounted for a sensitivity of 95% and a specificity of 82%. In terms of predictive values, the probability of truly presenting the SNV variant when it is detected by the nCounter panel was 82%, whereas the probability of not presenting the SNV variant when it is not detected by the platform was 95%. Finally, Cohen’s Kappa coefficient was 0.76, indicating a substantial correlation grade between OST and Vantage panels. Our results make nCounter an analytically sensitive, practical and cost-effective tool.

## 1. Introduction

Lung cancer is the leading cause of cancer worldwide, being responsible for more than 1.6 million deaths per year; more than breast, colon and prostate cancers combined [1]. During the last decades, comprehensive molecular profiling of non-small cell lung cancer (NSCLC) has revealed new actionable driver alterations that represent potential targets for inhibition. Such discoveries have changed the treatment paradigm giving rise to targeted therapies, which are systemic treatments that work by specifically blocking certain aspects of signaling pathways associated with tumour growth and suppression. Adenocarcinoma (ADC) is the lung cancer subtype that has most benefited from these molecular-based strategies due to its estimated frequency of oncogenic drivers higher than 60% [2].

Currently, several targeted therapies have been approved for the treatment of identified mutations in the *EGFR* [3,4,5], *BRAF* [6], or *MET* genes [7,8], as well as for gene rearrangements of *ALK* [9,10,11,12,13], *ROS1* [14,15], *NTRK* [16,17], and more recently, *RET* [18,19]. Therefore, several guidelines endorse routine genetic testing of the five leading oncogenes *EGFR*, *ALK*, *ROS1, BRAF* and *NTRK* in newly metastatic non-squamous NSCLC patients, while other molecular targets, such as *HER2* and *KRAS* mutations are approaching clinical practice [20,21,22]. *RET* rearrangements and *MET* exon 14 skipping (*MET*Δex14) mutations hopefully will be added soon to the list of mandatory testing genes.

Samples used for patient diagnosis frequently have limited tumour material and must be processed ensuring that the material is used sparingly, since many diagnostic tests may be required [23]. Therefore, the increasing necessity of testing more than one gene at a time has fostered the development of alternative detection modalities allowing testing for several biomarkers at the same time. In this context, highly efficient next-generation sequencing (NGS) technologies have allowed for a blanket testing of diverse potentially actionable driver genes from a single sample, being more efficient than their predecessor, Sanger sequencing [24]. NGS is based on the amplification and sequencing of DNA, allowing for the analysis of genomic regions and all the variants comprised in them in a reduced time and with low amounts of DNA that can be extracted from formalin-fixed paraffin-embedded (FFPE) and cytology samples. So far, there have been different validated NGS panels for the in vitro diagnosis of single nucleotide variants (SNVs), such as the Oncomine Solid Tumour (OST) panel DNA kit (ThermoFisher Scientific, Waltham, MA, USA) [25], and they represent the routine diagnostic tool in many hospitals [21]. However, there are other providers with different platforms offering multiple cancer panels for SNVs, such as Nanostring Technologies Inc (Seattle, WA, USA).

The nCounter Vantage 3D SNV Solid Tumour (Vantage) Panel from Nanostring Technologies is a new generation strategy based on direct hybridization of the DNA with different probes. Its lower DNA input requirements and short three-day turnaround compared to OST panel by NGS are features that make the Vantage panel a very attractive technique for daily clinical use. In our institution we already have experience working with the nCounter detection system. In a previous publication [26], we could retrospectively validate a multiplexed RNA-based nCounter codeset for the detection of *ALK*, *ROS1*, and *RET* fusion transcripts in FFPE samples from patients with advanced NSCLC, proving its advantage compared to standard diagnostic assays, such as fluorescent in situ hybridization (FISH) and immunohistochemistry (IHC). This RNA-based nCounter codeset has been complemented with other clinical-relevant genes and is currently being used in the routine clinical context of our institution to detect specific fusion transcripts—7 *ALK*, 10 *ROS1*, 6 *RET* and 2 *NTRK1*—and *MET*Δex14 mutations, giving significant good results in the context of molecular characterization of advanced NSCLC patients [27].

Herein, by using a set of FFPE samples obtained from advanced NSCLC patients, we aim to prospectively investigate the performance of the Vantage panel in the routine clinical context. We also aim to validate and compare the Vantage panel outputs with the OST panel in order to test whether the nCounter technique is a practical, reliable and sensitive method to identify the most meaningful somatic mutations associated with advanced lung ADC.

## 2. Materials and Methods

### 2.1. Patient Samples

Between January 2019 and November 2019, patients with advanced non-squamous NSCLC at our institution submitted to NGS analysis and with spare FFPE tumour material and/or cytological material were prospectively included in our study. Samples were obtained with the ethical committee approval at Hospital Clínic Barcelona (HCB/2017/1011; 13 February 2018) and prior full informed patient consent. The study was conducted in accordance with the principles of the Declaration of Helsinki.

Inclusion criteria were patients with non-squamous NSCLC with ADC histology and advanced stage. Exclusion criteria included those samples with insufficient material or DNA content for analysis.

### 2.2. DNA Purification and Quantification

FFPE slides (4 µm) or cytological smears (1 extension) were obtained by standard procedures and stained with hematoxylin and eosin. Tumour areas and the percentage of tumour infiltration were evaluated by a pathologist.

DNA isolation was performed with FFPE samples slides and cytological smears using the high-purity FFPE DNA isolation kit (QIAamp DNA FFPE Tissue Kit, QIAGEN, Hilden, Germany) following the manufacturer’s instructions. After extraction, DNA concentration was determined using Qubit Double Strand DNA High Sensitivity Assay Kit and Qubit Fluorometer (ThermoFisher Scientific, Waltham, MA, USA).

### 2.3. nCounter Vantage 3D Single Nucleotide Variant (SNV) Solid Tumour Panel Assay

After DNA extraction a multiplex polymerase chain reaction (PCR) amplification process was required to enrich targeted regions. DNA products were subsequently overnight hybridized directly with a multiplexed mixture of tags and reporter probes complementary to 96 SNVs, multinucleotide variants (MNVs), and indel variants associated with solid tumor biology from Nanostring Technologies. Variants can be detected as long as they are present at an allele frequency of 5% or greater. The nCounter codeset used allowed the detection of the variants by their binding to three different probes: S, T and M. Samples were subsequently processed in the nCounter Analysis System. Data collection was achieved by taking images of the immobilized fluorescent reporters in the sample cartridge with a CCD camera through a microscope objective lens. The number of images taken corresponded to the number of reporters counted. Finally, results were directly downloaded from the digital analyzer in RCC files format.

### 2.4. Oncomine Solid Tumour Panel DNA Kit

The NGS technique was performed following the manufacturer’s instructions, as described previously [27]. Twenty-two genes are represented in the panel and SNV variants were considered positive when present at an allele frequency of 5% or greater.

### 2.5. Data Analysis

Descriptive statistics, including the median and range for continuous variables and the percentages and frequencies for categorical variables, were tabulated and presented.

Results from the nCounter SNV Solid Tumour Panel assay were analyzed using nSolver v. 4.0 software by Nanostring Technologies. Data normalization was performed by using a Reference Run data set previously provided by Nanostring Technologies. Detection threshold was stablished at Log2 ratio relative to reference (Log2FC) >2. A *p*-value lower than 0.01 was considered for statistical significance. SNVs were reported independent of their clinical relevance.

Regarding the molecular diagnosis of the 41 patients submitted to both NGS and nCounter testing, diagnostic correlation was studied by classifying patients into three different categories: complete correlation, partial correlation and no correlation. Complete correlation was assumed when a determined SNV was identified by both platforms or when no mutation was detected; partial correlation corresponded to patients in which a SNV was detected by one platform but could not be assessed by the other technique since it was not included in the panel; finally, patients were classified as “no correlation” when significant diagnostic discrepancies appeared between techniques. Patients in which a mutation was detected by both platforms but who presented additional mutations included only in the OST or the Vantage panel were considered as complete correlation.

Sensitivity, specificity, positive predictive value (PV+) and negative predictive value (PV−) of the Vantage panel were assessed using standard methods. Correlation between results obtained with NGS and nCounter was determined by Cohen’s Kappa coefficient.

## 3. Results

### 3.1. Patient Cohort and Clinical Data

**Figure 1:**
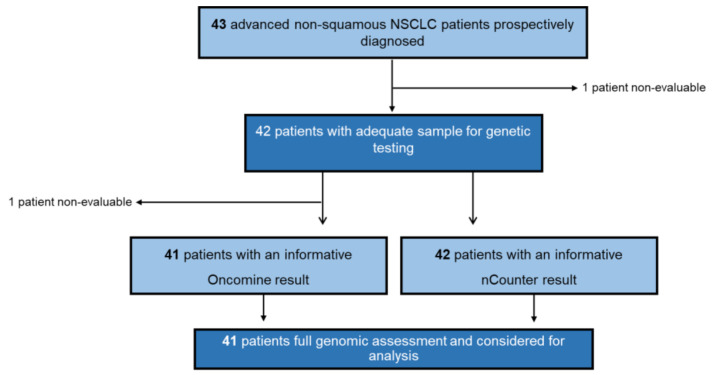
Flow diagram. NSCLC: non-small-cell lung cancer.

Between January 2019 and November 2019, a total of 43 advanced NSCLC patients were prospectively submitted to both NGS (OST) and nCounter (Vantage) DNA testing at our institution. Availability of FFPE block and hematoxylin and eosin stained samples was previously reviewed. Only one patient was excluded from both NGS and nCounter testing due to insufficient sample material. Regarding NGS, one additional patient (Patient 21) could not be finally tested due to the low quality of its DNA. DNA analysis by OST and/or Vantage yielded an informative result in 42 patients (98%). Among them, 41 (95%) had both NGS and nCounter informative results (Figure 1). All successfully genotyped patients had available clinical data.

Primary patient characteristics are shown in Table 1. Median age at diagnosis was 68 years (interquartile range of 50–86) and 74% of patients were men. Most patients (86%) were former or current smokers and 60% had an Eastern Cooperative Oncology Group (ECOG) performance status of 0 or 1 at disease diagnosis. The 95% of patient samples corresponded to primary tumours, while the two remaining cases represented two metastatic sites of lung ADC.

### 3.2. Molecular Characterization of Patients Using nCounter Vantage 3D SNV Solid Tumour Panel Assay

Among the 42 patients with adequate samples for testing, biopsies represented 79% (*n* = 33) of patient samples whereas cytologies were used in 21% (*n* = 9) of cases. Results of the DNA purification and quantification revealed that a minimum 5% of tumour content and a concentration of 5ng were sufficient to perform the nCounter Vantage 3D Solid Tumour Assay.

Genetic alterations were recorded in 23 patients (55%) using the Vantage panel. A total of 17 SNVs in 10 different genes were detected, being some of them concomitant. Driver alterations were found in 18 patients (43%), with *KRAS* (*n* = 9, 21%) and *EGFR* (*n* = 8, 19%) being the most commonly detected. Other less common driver alterations identified were *BRAF* and *PIK3CA*. Mutations in driver oncogenes were mutually exclusive except for two cases. In Patient 1 nCounter detected both *EGFR* E746_T751>VA and *PIK3CA* E542K mutations, however normalized data value (Log2FC) for the *EGFR* mutation was higher than that from the *PIK3CA*. For Patient 38, both *KRAS* G12D and *PIK3CA* E545K mutations were identified; in this case *KRAS* Log2FC was also higher than that of *PIK3CA*. Graphical representation of Patient 1 and Patient 38 results can be found in Figure S1.

**Figure 2:**
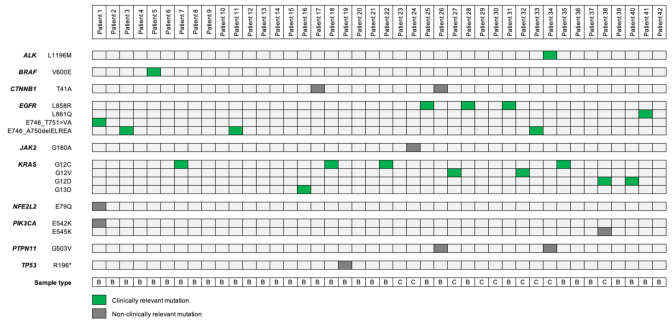
Mutation plot of single nucleotide variants identified in the 42 patients tested by the nCounter Vantage 3D Solid Tumour Assay. Clinically relevant stands for mutations which help to make clinical decisions, whether or not they can be targeted with a specific agent. B: Biopsy; C: Cytology.

Complete results from the 42 patients analyzed by the Vantage panel and nCounter Analysis System are shown in Figure 2.

**Figure 3:**
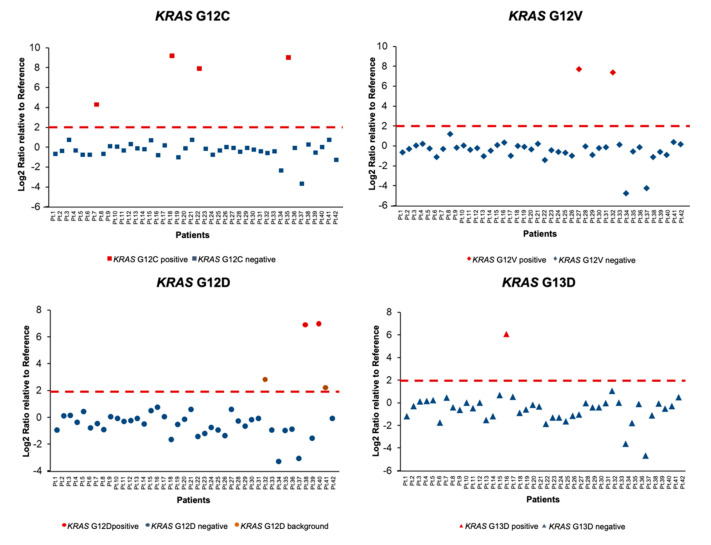
Normalized *KRAS* values for the 42 patients analyzed with nCounter platform. Pt: patient.

**Figure 4:**
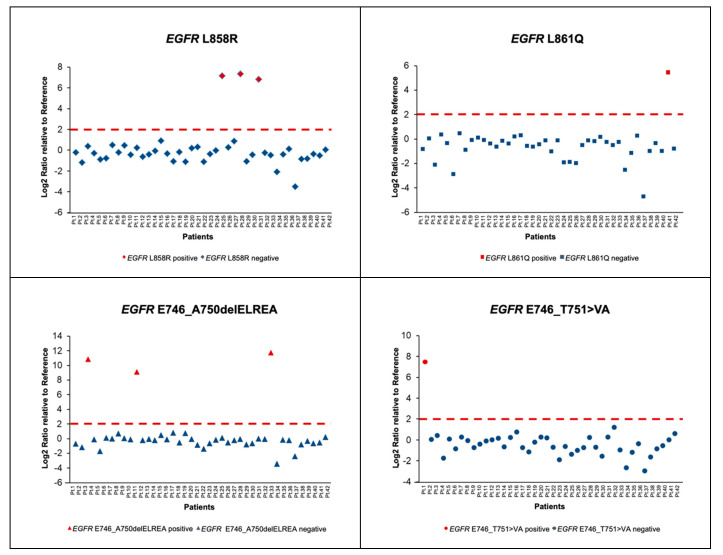
Normalized *EGFR* values for the 42 patients analyzed with nCounter platform. Pt: patient.

Overall, patients were considered positive for a given SNV when the Log2FC value was higher than 2. Focusing on *KRAS*, the most common driver alteration in our cohort, four distinct alterations (G12C, G12V, G12D and G13D) were found in nine patients with a median Log2FC of 7.4 (Figure 3). Of note, an additional *KRAS* G12D mutation was recorded in Patient 32 concomitantly with a *KRAS* G12V; however, owing to the difference between Log2FC values (2.8 for G12D vs. 7.8 for G12V), G12D was considered as background noise (Figure 3). In Patient 41, a *KRAS* G12D alteration was also classified as background noise; Log2FC value was 2.2 for *KRAS* mutation whilst an *EGFR* L861Q mutation with a Log2FC value of 5.5 was concomitantly reported (Figure 3). Regarding *EGFR*, we identified eight patients carrying four different mutations (L858R, L861Q, E746_A750delELREA and E746_T751>VA) and the median Log2FC was likewise 7.4 (Figure 4).

### 3.3. Comparison between Next-Generation Sequencing (NGS) (Oncomine Solid Tumor (OST) Panel) and nCounter Technology (Vantage Panel)

In terms of sample input, 5 ng of DNA were needed to perform the Vantage assay, whereas 10 ng of DNA were required to perform the OST panel focusing on hands-on time, nCounter required 30 min of benchwork every 12 samples, contrasting to the three to four hours required in order to perform a complete NGS analysis. Regarding execution time, on average two to four working days were sufficient to obtain the nCounter results, whereas 10 to 12 days were needed to dispose of NGS results.

**Figure 5:**
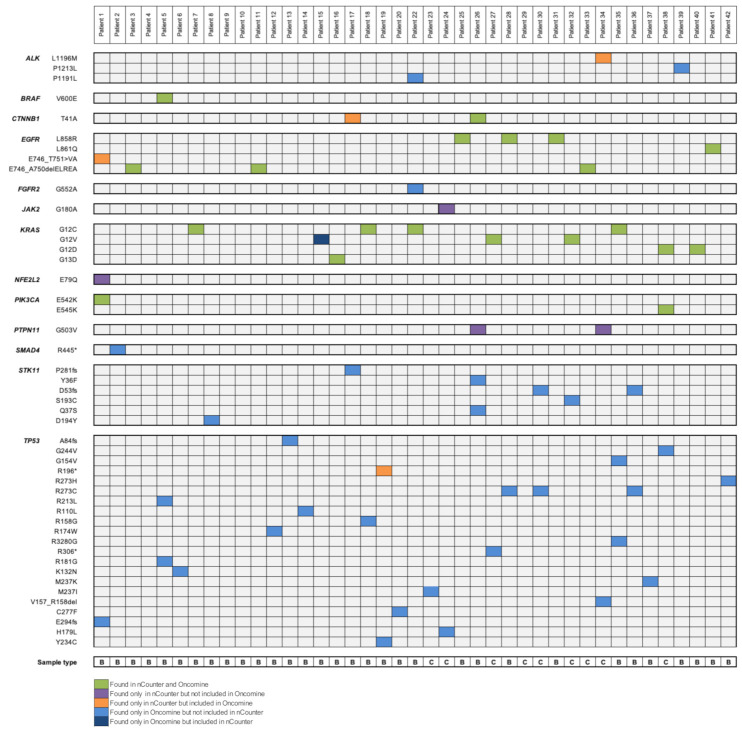
Mutation plot of single nucleotide variants identified in the 41 patients tested by both the nCounter Vantage 3D Solid Tumour Assay and the Oncomine Solid Tumour Panel DNA Kit. Alterations are colored depending in which panel they were detected. Four patients were negative for all the mutations tested. B: Biopsy; C: Cytology.

A total of 47 different variants from 13 genes were noted in both panels. From these 13 genes, 9 of them were represented in both platforms, 3 could only be tested by the nCounter panel and 1 appeared uniquely in the OST. The complete list of genes included in each DNA panel can be found in Figure S2. Concerning SNVs, 14 of them can be identified by both tumor panels, 3 only by the nCounter panel and 30 by the OST. Of note, 67% of the variants exclusively represented in the OST panel are *TP53* mutations. Complete molecular results from the 41 patients analyzed by both the OST and the Vantage panel are shown in Figure 5.

Regarding the molecular diagnosis of the 41 patients submitted to both NGS and nCounter testing, 51% of patients (*n* = 21) showed a complete correlation, 37% (*n* = 15) a partial correlation and 12% (*n* = 5) no correlation between both panels. Four patients (10%) were negative for all the mutations tested in both methods. For these cases, DNA quality was correct and no mutations were detected by other molecular techniques such as FISH, IHC or RNA-nCounter, thus they represented true wild-type cases. Five discordant cases were reported, with a median tumoral content of 70%. In Patient 1, the Vantage panel was able to detect a specific *EGFR* exon 19 deletion not reported by the OST. Due to scarce sample material, no orthogonal technique could be performed to elucidate the presence of this alteration. However, in the same patient sample, the *PIK3CA* E542K mutation was successfully identified by both technologies. In other three patients, nCounter technology detected a mutation not reported by NGS, these alterations were *CTNNB1* T41A in Patient 17, *TP53* R196* in Patient 19 and *ALK* L1196M in Patient 34. By contrast, ThermoFisher’s system detected a *KRAS* G12V mutation in Patient 15 which was not reported by Nanostring’s platform. The *KRAS* discrepancy in Patient 15 was analyzed using the real-time PCR-based platform Idylla (Biocartis NV, Mechelen, Belgium) as an orthogonal technique. The result was favorable to the OST panel, being *KRAS* G12V a confirmed nCounter false negative result.

In accordance with the previous results, the Vantage panel accounted for a sensitivity of 95% and a specificity of 82%. In terms of predictive values, the probability of truly presenting the SNV when it was detected by the Vantage panel was 82%, whereas the probability of not presenting the SNV when it was not detected by the platform was 95%. Finally, Cohen’s Kappa coefficient was 0.76, indicating a substantial correlation grade.

## 4. Discussion

Advanced NSCLC treatment has experienced an important revolution during the last few decades thanks to the introduction of targeted therapies in clinical practice; biomarker-directed therapy has been demonstrated to improve a patient’s quality of life and treatment outcomes compared to traditional chemotherapy [28]. However, genetic testing upfront at diagnose is needed in order to select patients for these systemic treatments. This necessary requirement can suppose a drawback in certain cases because small biopsies are common in NSCLC and sometimes these samples contain little tumor cell content with which to perform molecular testing [23]. Thus, analytical techniques requiring little amounts of DNA as well as low-quality analytes are becoming a “must” in NSCLC genotyping.

In this study, we have demonstrated the practicability and reliability of the Vantage panel for the prospective molecular characterization of advanced non-squamous NSCLC patients in the clinical setting. The present Vantage panel is useful to detect SNVs at the DNA level, however, it should be complemented with an RNA-analysis technique in order to include gene fusions, gene overexpressions and *MET*Δex14. In this context, the validation and implementation of an RNA-based customized nCounter panel for fusions, overexpressions and skipping detection has already been performed by our team [26,27]. This need for complementation has been proven in our cohort since 14% (*n* = 3) of patients classified as negative for any mutation by DNA analysis were later diagnosed by RNA analysis with *MET*Δex14 (*n* = 1) or with *MET* overexpression (*n* = 2). Thus, the joint use of the former DNA and RNA panels would give a broader picture of each patient’s molecular landscape, helping to select the proper treatment strategy.

Herein, we have been able to characterize 42 patients at the DNA level using both biopsy and cytological samples. All patients submitted to nCounter testing were evaluable, thus reporting a failure rate of 0, while in the OST panel one patient (see Figure 1) could not be analyzed due to low DNA quality. As a limitation, in some patients two DNA extractions were needed in order to obtain enough material to perform both techniques, however both extractions were performed from the same sample. In terms of sample type, both biopsies and cytological specimens have been equally useful to detect SNVs, with no significant differences between their performance and results (88–89% of concordant cases between nCounter and OST panels in both sample types). To date, different studies have already proven the usefulness of cytologies for determining the status of *EGFR*, *KRAS* and *ALK* genes [29,30]. Herein we have been able to identify three *KRAS* mutations (two *KRAS* G12V, one *KRAS* G12D; Figure 5) and one *EGFR* E746_A750delELREA (see Figure 5) from cytological smears by both Vantage and OST panels, thus supporting the adequacy of cytology for molecular testing.

Regarding the SNV correlation analysis it is important to remark that the two studied panels, the Vantage and the OST, are not grounded in the same technique, since the first is based on direct hybridization of the DNA with different probes and the second on NGS. Thus, nCounter technology can only test for the concrete variants contained in the panel whereas OST tests for genomic regions and all the variants they comprise. Therefore, the Vantage panel is not designed to identify undescribed mutations but for diagnosis purposes based on already described point mutations. However, Nanostring Technologies’ panel is flexible and can be customized by including new specific probes for determined SNVs if required. This versatility is an important point since molecular testing requirements are rapidly evolving and every day more genes switch from the “should be tested” to the “have to be tested” list.

All genes represented in the Vantage panel are clinically relevant in several tumor types, although not every single gene included is relevant concretely for NSCLC: some of them are druggable, such as *EGFR* and *BRAF*; some have a potential for future targeted strategies, such as *KRAS* G12C [31], whereas others, such as *STK11* and *KEAP1* are clinically informative although cannot be targeted yet [32]. Hence, the Vantage panel seems to be more suitable for clinical routine diagnostic rather than for investigational purposes, whereas the OST kit includes more genes which are informative but not relevant for treatment decisions.

Both multiplex platforms can test the two most mutated genes in NSCLC ADC, which are *EGFR* and *KRAS*. In our cohort results aligned with real life, with *KRAS* (n = 9, 21%) and *EGFR* (n = 8, 19%) being the driver alterations most commonly detected. The incidence of *KRAS* mutations is lower than that reported in the overall population perhaps owing to the small number of patients included in the study in comparison with broader molecular characterization analyses [33,34,35]. However, *EGFR* incidence matches the one reported in a previous series by our group [27], being higher than the frequencies reported for other European and Spanish cohorts [34,35], but close to the series collected in the USA [33]. Identifying *EGFR* status is one of the first steps in advanced-stage NSCLC management, as molecular targeted therapy is the standard of care in first-line treatment for patients carrying identified driver mutations in this gene [20,21,22]. *EGFR* mutated patients can be treated with tyrosine kinase inhibitors (TKIs), achieving significant, good responses and quality of life. However, up to 25% of patients carrying *EGFR* mutations are not eligible for TKI therapy owing to rapid progression [36]. Hence, it is important to determine *EGFR* status rapidly at diagnosis because its status will determine treatment guidance and prognosis. In addition, it is very common that patients treated with TKIs develop resistance in a short period of time. At resistance, a new biopsy must be obtained from tumors, delaying even more the process of molecular diagnose. Thus, it is relevant to have fast, simple and effective diagnostical techniques at our disposal to help guide multiple biomarker-driven targeted therapies. In this context, biopsy and/or cytology analysis by the Vantage panel would suppose an improvement as it requires only from two to four working days to obtain the results, with almost half of the time consumed by the OST panel. Regarding sample input, Nanostring technology required only 5ng of DNA, while exactly the double amount of nucleic acid (10 ng) was required for NGS.

There are some differences among genes represented in both panels. As previously mentioned, most relevant mutated genes in lung ADC are represented in both multiplex platforms, however, other relevant genes can only be tested in one of them. For instance, *ROS1* resistance mutation G2032R status is only included in the Vantage panel, providing the opportunity to identify patients which have become refractory to drugs currently approved for *ROS1* treatment and opening a window to the use of new therapies which could overcome this resistance [37]. Likewise, *JAK2* and *KIT* genes are uniquely represented in the Vantage panel. Although no specific targeted drugs have been approved for lung cancer, drugs such as cabozantinib, axitinib or regorafenib have been approved for other tumors harboring *KIT* mutations [38]. Once again, the nCounter technology would be helpful in guiding clinical management of patients. By contrast, all genes appearing in the OST panel but not in the Vantage are not druggable in the NSCLC context and only *MAP2K* can be targeted with selumetinib, a drug that seems to have promising efficacy in combination with docetaxel in *KRAS-*mutated NSCLC [39,40].

Five discordant cases were reported; however, discrepancies could only be tested by an orthogonal technique in one case due to insufficient sample material in the rest of cases. In this case, nCounter yielded a false negative result as orthogonal testing confirmed a *KRAS* G12V mutation successfully identified by the OST panel (see Figure 5). Another controversial case is *EGFR* E746_T751>VA alteration. In this case, the Vantage panel successfully identified this rare *EGFR* deletion, which was not otherwise reported by the OST panel. Due to the scarce material, no orthogonal testing could be performed. However, nCounter values for this mutation were significant and higher than the established cutoff threshold of positivity (Log2FC = 7.6, cutoff Log2FC >2). Moreover, in this case two sequential DNA extractions from the same sample were needed in order to obtain sufficient DNA material to perform both the Vantage and the OST panels; thus, tumoral heterogeneity could be the reason for the discrepancy.

To the best of our knowledge, this is the first study using the nCounter Vantage 3D SNV Solid Tumor Panel from Nanostring Technologies in a prospective manner in the routine workup of advanced non-squamous NSCLC patients. The correlation analysis has revealed an important 88% agreement between Vantage and OST results, with a Cohen’s Kappa coefficient of 0.76 indicating a substantial correlation grade. Sensitivity (95%), specificity (82%) and positive and negative predictive values observed (82% and 95%, respectively) make the Vantage panel an analytically sensitive, practical and cost-effective tool.

## Figures and Tables

**Table 1:** IQR: interquartile range.

Variables	Total *n*. (%) (*n* = 43)
Age at diagnosis, median (IQR)	68 (50–86)
Sex	
Women	11 (26)
Men	32 (74)
Sample Site	
Primary tumour	41 (95)
Metastasis	2 (5)
Source of Material	
Biopsy	33 (77)
Citology	10 (23)
Performance Status ^1^	
0	10 (23)
1	16 (37)
2	8 (19)
3	5 (12)
4	0 (0)
Unknown	4 (9)
Smoking History	
Never	5 (12)
Former ^2^	20 (46)
Current	17 (40)
Unknown	1 (2)

^1^ The ECOG (Eastern Cooperative Oncology Group) performance status of 0 (fully active); 1 (restricted in physically strenuous activity, but ambulatory and able to carry out work of a light or sedentary nature); 2 (ambulatory and capable of all self-care, but unable to carry out any work activities; up and about more than 50% of waking hours); 3 (capable of only limited self-care; confined to bed or chair more than 50% of waking hours); and 4 (completely disabled). ^2^ Patients who have not smoked for the last year or more.

## Supplementary Materials

The following are available online at https://www.mdpi.com/2075-4418/10/11/902/s1, Figure S1: Patient 1 and Patient 38 co-mutation plots; Figure S2: nCounter Vantage 3D Solid Tumor Assay panel and Oncomine Solid Tumor Panel DNA Kit content. The genes included in each panel are colored green.
