# Supplementary material for: Prospective Evaluation of Single Nucleotide Variants by Two Different Technologies in Paraffin Samples of Advanced Non-Small Cell Lung Cancer Patients
Source: Diagnostics (Basel). 2020 Nov 3;10(11):902. doi: 10.3390/diagnostics10110902 (PMC7693424; doi:10.3390/diagnostics10110902)
Supplement: Supplementary file 1 [file diagnostics-10-00902-s001.pdf]

Supp. Figure 1. SNV COMUTATION CALL EXAMPLE (Pt.1)

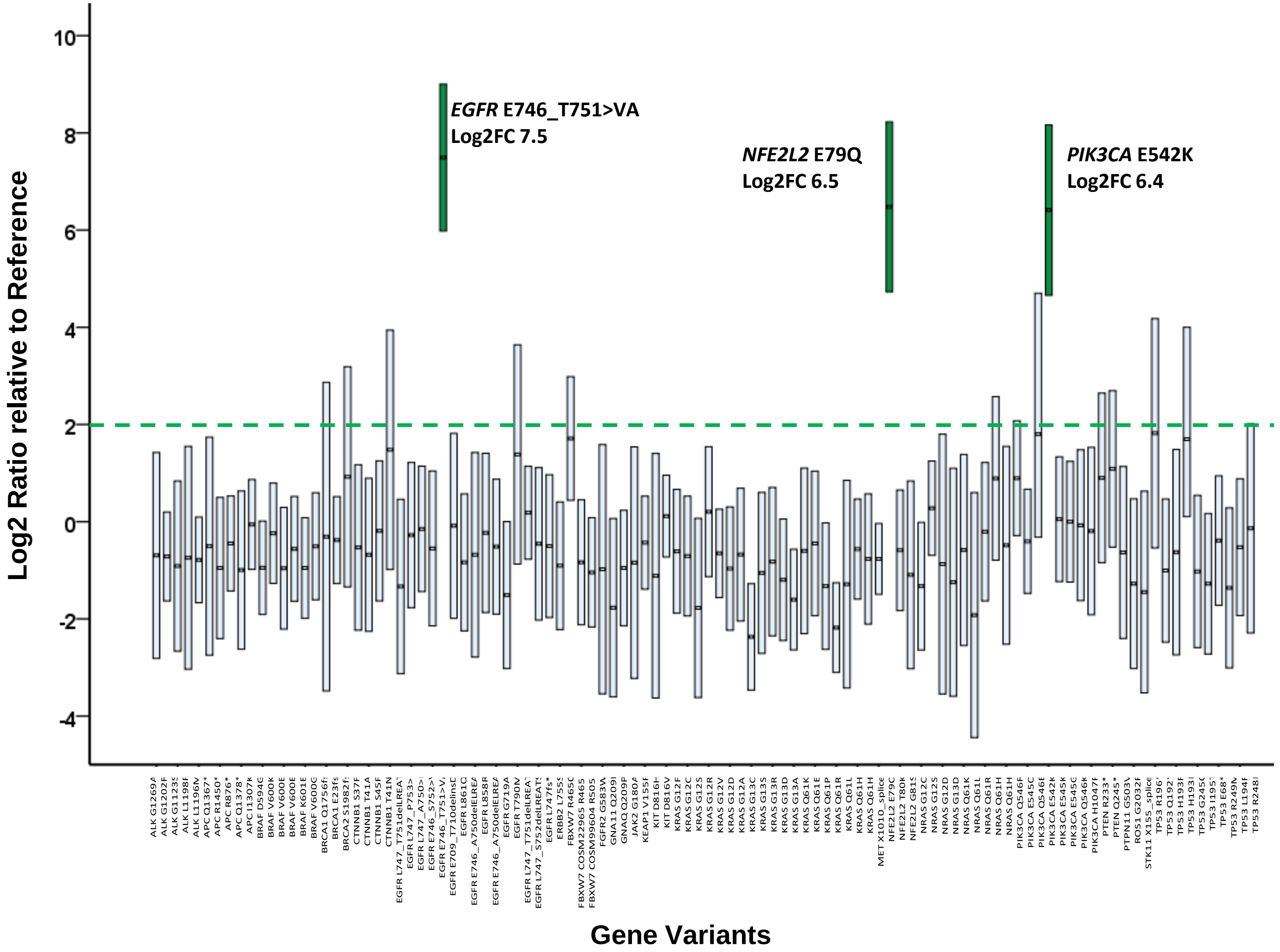

Mutation undetected: log2(fc)< 1 or raw count< 100 and 0.01< p value

Mutation detected and clinically significant: 2< log2(fc) and 200< raw count and p value <0.01

Supp. Figure 1. SNV COMUTATION CALL EXAMPLE (Pt.38)

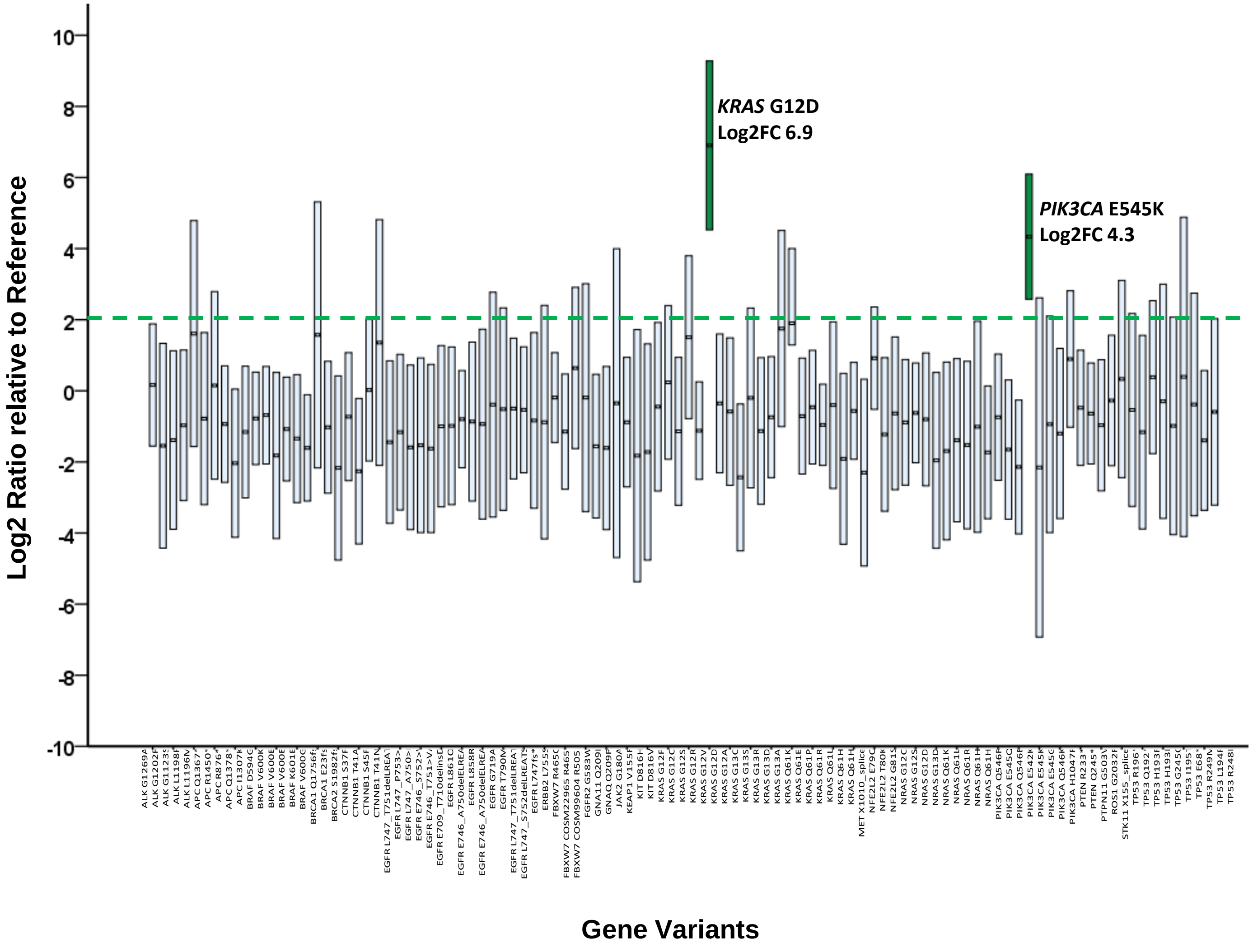

Mutation undetected: log2(fc)< 1 or raw count< 100 and 0.01< p value

Mutation detected and clinically significant: 2< log2(fc) and 200< raw count and p value <0.01

Supp. Figure 2. nCounter Vantage 3D Solid Tumor Assay panel and Oncomine Solid Tumor Panel DNA Kit content

Gene	nCounter Vantage 3D SNVSolid Tumor Panel	Oncomine Solid Tumor DNA Panel
ALK		
AKT1		
APC		
BRAF		
BRCA1		
BRCA2		
CTNNB1		
DDR2		
EGFR		
ERBB2		
ERBB4		
FBXW7		
FGFR1		
FGFR2		
FGFR3		
GNA11		
GNAQ		
JAK2		
KEAP1		
KIT		
KRAS		
MAP2K1		
MET		
NFE2L2		
NOTCH1		
NRAS		
PIK3CA		
PTEN		
PTPN11		
ROS1		
SMAD4		
STK11		
TP53		
